# Human Infections with New Subspecies of *Campylobacter fetus*

**DOI:** 10.3201/eid1910.130883

**Published:** 2013-10

**Authors:** Mary E. Patrick, Maarten J. Gilbert, Martin J. Blaser, Robert V. Tauxe, Jaap A. Wagenaar, Collette Fitzgerald

**Affiliations:** Centers for Disease Control and Prevention, Atlanta, Georgia, USA (M.E. Patrick, R.V. Tauxe, C. Fitzgerald);; Utrecht University, Utrecht, the Netherlands (M.J. Gilbert, J.A. Wagenaar);; New York University School of Medicine, New York, New York, USA (M.J. Blaser);; Central Veterinary Institute, Lelystad, the Netherlands (J. Wagenaar);; World Health Organization Collaborating Center for Campylobacter/World Organisation for Animal Health Reference Laboratory for Campylobacteriosis, Utrecht (J.A. Wagenaar, M.J. Gilbert)

**Keywords:** Campylobacter fetus, C. fetus subsp. fetus, C. fetus subsp. venerealis, C. fetus subsp. testudinum subsp. nov., bacteria, reptiles, epidemiology, foodborne infections, human infections

## Abstract

*Campylobacter fetus* subsp. *testudinum* subsp. nov. is a newly proposed subspecies of *C. fetus* with markers of reptile origin. We summarize epidemiologic information for 9 humans infected with this bacterium. All cases were in men, most of whom were of Asian origin. Infection might have been related to exposure to Asian foods or reptiles.

*Campylobacter* spp. are the most common cause of bacterial gastrointestinal illness in humans. *C. jejuni* is the most common species and accounts for >88% of reported cases in the United States (*1*). *C. fetus* is an uncommonly reported species that typically affects `immunocompromised, pregnant, or elderly persons and causes severe infections, including bacteremia and meningitis (*2*).

Two subspecies of *C. fetus* have been described: *C. fetus* subsp. *fetus* and *C. fetus* subsp. *venerealis* (*3*). *C. fetus* subsp. *fetus* has been isolated from intestinal tracts of sheep and cattle and from tissues from sporadic abortions in these species. *C. fetus* subsp. *venerealis* is restricted to cattle and causes bovine genital campylobacteriosis (*4*). Although *C. fetus* subsp. *venerealis* has been isolated from humans (*5*), its role in human disease is uncertain; most cases of *C. fetus* infection are caused by *C. fetus* subsp. *fetus*.

In 1984, *C. fetus* was isolated from feces of a reptile, a Florida box turtle (*Terrapene carolina bauri*) that was kept as a pet (*6*). *C. fetus* has also been isolated from feces of a healthy western hognose snake (*Heterodon nasicus*) and a blotched blue-tongue lizard (*Tiliqua nigrolutea*) that had unformed feces and was losing weight (*7*). Substantial genetic divergence between *C. fetus* strains of reptile and mammal origin has been demonstrated (*8*).

A human isolate of *C*. *fetus* with markers of reptile origin was reported in 2004 (*9*). A subsequent study involving phenotypic and molecular characterization of the 2004 human case, 4 additional human cases, and 3 reptiles definitively identified this collection of strains as a newly proposed subspecies named *C. fetus* subsp. *testudinum* subsp. nov. (*7**,**10*). The Centers for Disease Control and Prevention recently screened *Campylobacter* strains from its historical culture collection and identified 4 additional human cases of infection with this subspecies.

## The Study

We collected demographic and epidemiologic information to describe characteristics of the 9 reported patients infected with *C. fetus* subsp. *testudinum* subsp. nov. Food preferences and limited information about exposures were available for 5 patients. Four patients could not be interviewed because they could not be located or had died. However, some information was available from their original case reports. We summarize our findings in the Table.

**Table:** Characteristics of 9 men with *Campylobacter fetus* subsp. *testudinum* subsp. nov. infection, 1991–2010*

Patient/age, y	Year	Ancestry	Isolate no.	Strain no.	Specimen source	Clinical symptoms	Underlying conditions	Hospitalized	Died	Food history	Animal contact
1/NA	1991	NA	D6783	91–2	Feces	NA	NA	NA	No	NA	NA
2/30	1992	NA	D4335		Feces	Diarrhea	NA	NA	NA	NA	Turtle
3/80	2002	NA	D6128		Blood	NA	NA	NA	NA	NA	NA
4/20	2003	Chinese	D6688, D6689†	03–427, 03–445	Blood	Fever, cough, epigastric pain	Leukemia, hepatitis B	Yes	Yes	Turtle soup, angelica herb	NA
5/79	2004	Chinese or Vietnamese	D6659		Pleural fluid	Bloody diarrhea, pulmonary edema	Liver cancer	Yes	NA	NA	No
6/84	2005	Chinese	D6683		Hematoma	Speech and motor difficulties, hematoma	Asthma	Yes	No	Chinese dishes, eel, frog	No
7/67	2005	Chinese	D6690	05–018	Blood	Fever, chills, rigor, cough, diarrhea	Lymphoma, hypertension, heart disease	Yes	No	Chinese dishes	No
8/85	2007	Chinese	D6856		Bile	Diarrhea	NA	Yes	NA	NA	No
9/62	2010	Caucasian‡	D9240		Blood	Cellulitis of leg	Diabetes	Yes	No	Chinese dishes, eel	No

*NA, not available. †Two isolates were obtained from this patient. ‡Asian spouse.

Patients resided in Colorado, Louisiana, Massachusetts, and New York, and had onset of illness during 1991–2010. All patients were men (median age 73 years, range 20–90 years). Five of 6 patients were of Asian origin (4 were Chinese and 1 was either Chinese or Vietnamese), and the non-Asian patient had a Chinese spouse. Last names of the remaining 3 patients did not suggest that they were of Asian origin.

*C. fetus* subsp. *testudinum* subsp. nov. was isolated from blood (4 patients), feces (2 patients) pleural fluid (1 patient), hematoma (1 patient), and bile (1 patient). Of 5 patients with available information, all had underlying illness. Clinical symptoms varied. One patient had fever, cough, and epigastric pain; another had fever, chills, rigors, cough, and diarrhea; and a third had bloody feces, pulmonary edema, and pleural effusion. One patient sought care for dizziness and mental confusion after a fall, and *C. fetus* subsp. *testudinum* subsp. nov. was isolated from a subdural hematoma. For another patient, *C. fetus* subsp. *testudinum* subsp. nov. was isolated from blood after cellulitis developed from a leg wound; no gastrointestinal symptoms were reported. All 6 patients for whom outcomes were available were hospitalized, and 1 died of leukemia.

All 5 patients of Asian origin and the 1 patient with an Asian spouse shopped or ate at restaurants in Chinese (Chinatown) areas in Massachusetts and New York. A limited food and travel history was available for 4 patients. All 4 reported eating traditional Chinese dishes. In addition, 1 patient ate eel, 1 ate eel and frog, 1 ate turtle soup, and 1 denied eating turtle or frog. Three patients did not report any recent travel, and 1 reported frequent travel, including trips to Europe and Hong Kong. Food and travel histories were not available for the non-Asian patients. However, 1 patient reported contact with a turtle that had diarrhea. This patient did not appear to have had a systemic infection; his isolate was obtained from feces, and he reported a 16-day history of diarrhea.

## Conclusions

*C. fetus* subsp. *testudinum* subsp. nov. is a newly proposed subspecies that appears to have originated in reptile species. Although information is limited, our data suggest that humans may contract this subspecies though exposure to reptiles, possibly by ingestion or by contact with feces or the environment. Reptiles, particularly small turtles, are a well-known source of *Salmonella* spp. infections in humans (*11*). A recent study in Taiwan reported *C. fetus* subsp. *testudinum* subsp. nov. in feces of 12 (6.7%) of 179 reptile feces samples; prevalence was highest in turtles (10 [9.7%] of 103) (*12*). Turtle is an ingredient in some traditional Asian dishes and turtles are frequently sold in Asian specialty markets; this association might partly explain the predominance of Asian race among reported patients. A review of GenBank found 5 submissions of 16S rDNA sequences representing *C. fetus* subsp. *testudinum* subsp. nov. from China (accession nos. DQ997044, HQ450384, HQ681195, JN585921, and JN585922).

Although *C. fetus* is more common among men (*13**,**14*), it is unusual that all of the recognized patients infected with *C. fetus* subsp. *testudinum* subsp. nov. were men. This finding might indicate that men have a predisposition to infection with this subspecies or that they are more likely to be exposed to sources of contamination. Nearly all patients with *C. fetus* subsp. *testudinum* subsp. nov. infections were >60 years of age or had immunocompromising conditions. This finding indicates that *C. fetus* subsp. *testudinum* subsp. nov., like *C. fetus* subsp. *fetus*, are opportunistic pathogens that might lead to severe disease. Most patients had primary bacteremia; only 4 of 7 patients had diarrhea associated with their illnesses.

The actual number of *C. fetus* subsp. *testudinum* subsp. nov. illnesses is unknown. *C. fetus* infections are likely to be underdiagnosed and underreported. *C. fetus* is susceptible to cephalosporins, which are commonly included in media used for isolation of *Campylobacter* spp., making *C. fetus* isolation from feces unlikely. *Campylobacter* spp. infection is not a nationally reportable disease in the United States, and in most states, isolates are not routinely sent to state public health laboratories for confirmation of identification. Although some clinical and state laboratories identify *Campylobacter* spp., this identification is not conducted routinely, and few laboratories use molecular methods to identify strains to the species level. For *C. fetus* subsp. *testudinum* subsp. nov., additional identification methods have to be performed. Therefore, we encourage laboratories that identify the *Campylobacter* spp. to forward isolates of *C. fetus* to the *Campylobacter* Reference Laboratory at the Centers for Disease Control and Prevention for confirmation and screening for *C. fetus* subsp. *testudinum* subsp. nov. In addition, when interviewing persons with *Campylobacter* spp. infections, public health personnel should ask about exposure to live reptiles and traditional Asian dishes made with turtles or other reptiles.

In summary, our data show that *C. fetus* subsp. *testudinum* subsp. nov. can cause invasive infection. All known cases have occurred in men, most of whom were of Asian origin, and infection may be related to exposure to traditional Asian foods or reptiles. Persons who are immunocompromised should avoid eating undercooked reptiles, exposure to live reptiles, and their environments. Enhanced public health surveillance and laboratory testing and surveys of the prevalence of the organism in reptiles are needed to better understand the epidemiology and incidence of *C. fetus* subsp. *testudinum* subsp. nov. and to recommend additional prevention measures.
